# Metronidazole-Associated Peripheral Neuropathy in a Patient With End-Stage Renal Failure

**DOI:** 10.7759/cureus.75373

**Published:** 2024-12-09

**Authors:** Michelle Shin, Otarod Bahrani

**Affiliations:** 1 Internal Medicine, Eastern Virginia Medical School, Norfolk, USA

**Keywords:** metronidazole, neurotoxicity, peripheral neuropathy, renal dosing, renal failure

## Abstract

Metronidazole is a broad-spectrum antimicrobial that is associated with 0.16% 100-day incidence rates for peripheral neuropathy. In this report, we present an interesting and rare presentation of peripheral neuropathy secondary to metronidazole use for *Serratia* bacteremia and sepsis in a patient with end-stage renal failure.

## Introduction

Metronidazole is a broad-spectrum antimicrobial used to treat anaerobic bacterial, protozoal, and microaerophilic infections. It is FDA-approved for conditions like trichomoniasis, amebiasis, giardiasis, bacterial vaginosis, and various anaerobic infections (e.g., *Bacteroides* and *Clostridium* species). Additionally, it is used topically for rosacea and intravaginally for bacterial vaginosis, with off-label uses in conditions such as *Clostridioides* *difficile* infection, Crohn's disease, and perianal fistulas [1]. The drug works by disrupting DNA synthesis in microorganisms, with rapid bactericidal effects that are largely concentration-dependent. Metronidazole is well-absorbed and penetrates the blood-brain barrier, also making it effective for central nervous system (CNS) infections. 

Metronidazole is generally well-tolerated with mild to moderate side effects. The most commonly reported side effect is gastrointestinal symptoms including nausea, abdominal pain, and diarrhea. Over the years, there have been rare reports of neurotoxicity, i.e., peripheral neuropathy or convulsive seizures, with the use of metronidazole, though the mechanism by which this occurs is not fully understood. Cerebellar degeneration is likewise a known neurotoxic effect of metronidazole toxicity with a specific predilection to involve the dentate nucleus [2]. 

## Case presentation

The patient is a 62-year-old woman with a past medical history of type II diabetes mellitus and end-stage renal disease (ESRD). This patient had been receiving hemodialysis three times a week for the past eight years. Her medications include epoetin alfa (erythropoietin-stimulating agent) for anemia, B12 complex, and sevelamer with meals. Her diabetes regimen consisted of insulin lispro 2-10 unit injections before meals and nightly. Her diabetes was well-controlled at the time of initial admission. Her most recent HA1c was 6.9 with an estimated average blood glucose of 152 mg/dL. 

This patient had initially presented to the hospital with three-vessel coronary artery disease, mitral stenosis/regurgitation, and tricuspid regurgitation. The patient ultimately opted to proceed with coronary artery bypass grafting, mitral valve replacement, and tricuspid valve repair. This patient was ultimately admitted to the extended recovery unit to bridge her hospital stay with her eventual discharge to a skilled nursing facility. Her postoperative course was subsequently complicated by left lower extremity purpura and ulceration with eschar formation in the setting of critical limb ischemia due to peripheral artery disease. 

Overnight, this patient developed a fever of 101.6°F with an elevated white blood cell count of 20,000 (Table 1). She was unable to tolerate hemodialysis the night prior due to tachycardia and generally feeling unwell according to her nurses' report. On examination, the patient was alert and in no acute distress, but did appear more fatigued than her baseline. Her wound culture grew *Serratia*, and she was found to have gram-negative rod bacteremia due to acalculous cholecystitis. Interventional radiology placed a percutaneous cholecystostomy drain. Infectious disease was consulted for gram-negative rod bacteremia, and she was subsequently started on intravenous ceftriaxone 2 g every 24 hours for 10 days and metronidazole 500 mg by mouth every 12 hours for five days.

**Table 1:** Patient lab findings BUN: blood urea nitrogen; AST: aspartate aminotransferase; ALT: alanine aminotransferase

	Patient labs	Normal range [3]
White blood cell count	20,000	4,500-11,000
Hemoglobin	8.0	12.0-16.0
Hematocrit	27.2	36-46%
Mean corpuscular volume	105	80-100
Mean corpuscular hemoglobin concentration	29	31-36%
Red cell distribution width	24.3	12-15%
Platelets	408,000	150,000-400,000
Sodium	131	136-146
Potassium	4.8	3.5-5.0
Chloride	93	95-105
Carbon dioxide	19	22-28
Anion gap	19	4-12
BUN	44	7-18
Creatinine	5.7	0.6-1.2
Glucose	158	<140
Calcium	10.0	8.4-10.2
Phosphorus	5.0	3.0-4.5
Albumin	3.2	3.5-5.5
Total protein	6.3	6.0-7.8
Alkaline phosphatase	121	25-100
AST	21	12-38
ALT	14	10-40
Total bilirubin	0.4	0.1-1.0

On day 3 of antibiotic therapy, she began to complain of neuropathic symptoms, namely, numbness and tingling in her hands with the right being worse than the left. She described that she had dropped her television remote from her right hand several times over the past day due to reduced strength. During the neurologic examination, she was alert and oriented to person, place, and time. Her extraocular movements and all 12 cranial nerves were intact. She did have notably reduced strength in both hands, 1/5 on her right and 2/5 on her left. Additionally, she was tender to palpation over the distal interphalangeal joints and the pads of the first to third digits of her right hand. Strength and sensation were intact and symmetric in her proximal upper extremities as well as her lower extremities. 

The decision was made to discontinue metronidazole given her peripheral neuropathy. There was almost immediate improvement in her symptoms within 24 hours. By the next morning, she reported a subjective reduction in her hand numbness and weakness. Her exam showed improved grip strength, 2/5 on her right and 3/5 on her left. She endorsed minimal tenderness to palpation over her fingertips. Subsequently, within three days of discontinuing metronidazole, there was almost complete resolution of her peripheral neuropathy. Her hands had both returned to baseline strength 5/5 and she reported no numbness or tingling.

## Discussion

Metronidazole-associated peripheral neuropathy is rare and has only been reported in under 40 patients in the current literature. One systematic review done in 2018 found that within clinical studies, there was a higher incidence of peripheral neuropathy in patients receiving >42 g total (>4-week period) of metronidazole compared with those patients receiving ≤42 g total (17.9% vs. 1.7%) [4]. In other words, there may be some relation to dose. Peripheral neuropathy is, thus, very rare in patients who receive ≤42 g total of metronidazole. 

For intra-abdominal infections, metronidazole dosing typically follows 7.5 mg/kg PO every six hours for 7-10 days [1]. Given this patient's clinical course, complex past medical history, and relatively immunocompromised status with her advanced age and ESRD, the decision was made to proceed with a shorter, higher-dosed course of metronidazole (500 mg twice daily for five days). Interestingly, because metronidazole is renally cleared, it is possible that her end-stage renal failure played a role in developing transient metronidazole toxicity despite receiving a much lower total dose than what has been found to cause neurotoxicity in current literature. In fact, one case-control study showed that patients with concomitant renal disease were at particularly high risk for these neuropathic complications secondary to metronidazole use [5]. 

Of the known reports of metronidazole-associated peripheral neuropathy, nearly all patients had complete resolution of symptoms upon discontinuation. Patients who receive higher total doses may be at higher risk of peripheral neuropathy, but our patient poses an interesting case in which a very low total dosage of metronidazole resulted in neurotoxicity. We considered other causes of acute peripheral neuropathy including Guillain-Barré syndrome (GBS), vitamin deficiencies, uremia, and acute nerve compression. However, given this patient was actively receiving hemodialysis treatment, uremia was less likely. There was no evidence of trauma that would point to nerve injury. GBS was less likely due to the absence of clinical symptoms. This patient did not have ascending symptomatology, and there was no involvement of the lower limbs; therefore, this patient's clinical presentation did not warrant electromyography (EMG) evaluation. 

The mechanism of metronidazole-induced peripheral neuropathy remains unclear, but several mechanisms have been proposed. Proposed mechanisms include the binding of metronidazole to RNA, DNA, and inhibitory neurotransmitters, as well as inducing both vasogenic and cytotoxic edema, and colleagues suggested that nerve damage is the result of free radicals produced during metronidazole metabolism [6-9]. Resultant neurotoxicity may present as isolated peripheral neuropathy or in combination with other symptoms related to cerebellar toxicity, including gait disturbances, weakness, confusion, dysarthria, ataxia, dysmetria, and dysphagia [10].

## Conclusions

To our knowledge, this is a rare report about a case of metronidazole-associated peripheral neuropathy in a patient with end-stage renal failure. EMG remains an excellent diagnostic tool to assess the extent, severity, and prognosis of complex regional pain syndrome (CRPS) in this case. Currently, there are no recommendations to renally dose metronidazole in patients with ESRD. However, patients like ours add to a small body of case-based evidence to support conservative dosing and closer monitoring of patients with a history of renal disease.
